# Deep learning–based dose prediction to improve the plan quality of volumetric modulated arc therapy for gynecologic cancers

**DOI:** 10.1002/mp.16735

**Published:** 2023-09-14

**Authors:** Mary P. Gronberg, Anuja Jhingran, Tucker J. Netherton, Skylar S. Gay, Carlos E. Cardenas, Christine Chung, David Fuentes, Clifton D. Fuller, Rebecca M. Howell, Meena Khan, Tze Yee Lim, Barbara Marquez, Adenike M. Olanrewaju, Christine B. Peterson, Ivan Vazquez, Thomas J. Whitaker, Zachary Wooten, Ming Yang, Laurence E. Court

**Affiliations:** ^1^ Department of Radiation Physics The University of Texas MD Anderson Cancer Center Houston Texas USA; ^2^ The University of Texas MD Anderson Cancer Center UTHealth Houston Graduate School of Biomedical Sciences Houston Texas USA; ^3^ Department of Radiation Oncology The University of Texas MD Anderson Cancer Center Houston Texas USA; ^4^ Department of Radiation Oncology The University of Alabama at Birmingham Birmingham Alabama USA; ^5^ Department of Imaging Physics The University of Texas MD Anderson Cancer Center Houston Texas USA; ^6^ Department of Biostatistics The University of Texas MD Anderson Cancer Center Houston Texas USA; ^7^ Department of Statistics Rice University Houston Texas USA

**Keywords:** artificial intelligence, deep learning, dose prediction, quality assurance

## Abstract

**Background:**

In recent years, deep‐learning models have been used to predict entire three‐dimensional dose distributions. However, the usability of dose predictions to improve plan quality should be further investigated.

**Purpose:**

To develop a deep‐learning model to predict high‐quality dose distributions for volumetric modulated arc therapy (VMAT) plans for patients with gynecologic cancer and to evaluate their usability in driving plan quality improvements.

**Methods:**

A total of 79 VMAT plans for the female pelvis were used to train (47 plans), validate (16 plans), and test (16 plans) 3D dense dilated U‐Net models to predict 3D dose distributions. The models received the normalized CT scan, dose prescription, and target and normal tissue contours as inputs. Three models were used to predict the dose distributions for plans in the test set. A radiation oncologist specializing in the treatment of gynecologic cancers scored the test set predictions using a 5‐point scale (5, acceptable as‐is; 4, prefer minor edits; 3, minor edits needed; 2, major edits needed; and 1, unacceptable). The clinical plans for which the dose predictions indicated that improvements could be made were reoptimized with constraints extracted from the predictions.

**Results:**

The predicted dose distributions in the test set were of comparable quality to the clinical plans. The mean voxel‐wise dose difference was −0.14 ± 0.46 Gy. The percentage dose differences in the predicted target metrics of $D_{1\%}$ and $D_{98\%}$ were −1.05% ± 0.59% and 0.21% ± 0.28%, respectively. The dose differences in the predicted organ at risk mean and maximum doses were −0.30 ± 1.66 Gy and −0.42 ± 2.07 Gy, respectively. A radiation oncologist deemed all of the predicted dose distributions clinically acceptable; 12 received a score of 5, and four received a score of 4. Replanning of flagged plans (five plans) showed that the original plans could be further optimized to give dose distributions close to the predicted dose distributions.

**Conclusions:**

Deep‐learning dose prediction can be used to predict high‐quality and clinically acceptable dose distributions for VMAT female pelvis plans, which can then be used to identify plans that can be improved with additional optimization.

## 1 INTRODUCTION

External beam radiotherapy is a common treatment for gynecologic cancers and is often followed by intracavitary brachytherapy. With technological innovations, external beam treatment techniques for the female pelvis have advanced from two‐dimensional four‐field‐box techniques to three‐dimensional (3D) conformal radiotherapy techniques and intensity‐modulated radiotherapy (IMRT) techniques. With IMRT, the radiation dose can be modulated to achieve superior plans with conformal target doses and reduced doses to normal tissue. IMRT plans are created using inverse treatment planning, in which a planner creates a list of planning objectives which are used to build the objective function that is optimized to find acceptable plans. The IMRT plan quality is highly dependent on the skill of the planner,^1^ the expectations of the attending radiation oncologist,^2^ the outcomes of any peer review processes.^3^ While scorecards exist to ensure that the target and normal tissue constraints are met, a skilled planner/oncologist team has the expertise to know when further normal tissue sparing is achievable based on patient and tumor characteristics.

Geometric models that correlate patient geometry with achievable dose‐volume histograms (DVHs)^4^, ^5^, ^6^, ^7^, ^8^ are advantageous for patient‐specific treatment plan quality assurance.^9^, ^10^, ^11^ With the advent of deep learning, more advanced models that enable the prediction of entire 3D dose distributions have been developed in recent years.^12^, ^13^, ^14^, ^15^, ^16^, ^17^, ^18^, ^19^, ^20^, ^21^, ^22^, ^23^, ^24^, ^25^, ^26^, ^27^, ^28^, ^29^, ^30^, ^31^, ^32^, ^33^ The potential of deep learning–based dose prediction to develop patient‐specific plan quality assurance tools should be further investigated. In our prior work, we showed the ability of deep learning–based dose predictions to automatically identify suboptimal head and neck plans.^2^ Here, we have expanded on our prior work in artificial intelligence–based plan quality assurance. Specifically, we have investigated the use of deep‐learning dose prediction to drive plan quality improvements for external beam radiotherapy for the treatment of gynecologic cancers. To date, 3D dose prediction for external‐beam radiotherapy of the female pelvis has not been widely studied.^27^, ^32^, ^33^

The purpose of this study was to predict high‐quality dose distributions for volumetric modulated arc therapy (VMAT) plans for patients with gynecologic cancers and to evaluate the usability of the predicted dose distributions in improving clinical plan quality. To the best of our knowledge, this is the first deep‐learning dose prediction study to include a physician review of the dose predictions and to test the achievability of the predicted dose distributions in cases in which the predictions showed higher quality distributions (i.e., more sparing of normal tissue) than the original plans. In this work, we demonstrate the potential of predicted dose distributions to both identify suboptimal clinical plans and guide plan re‐optimization to improve clinical plan quality. This is of particular importance, as it demonstrates that these tools can be used to identify improvements *and* drive change.

## 2 METHODS

### 2.1 Patient data and dose prediction methodology

The dataset consisted of 79 clinically approved VMAT plans with a single target dose level prescribed to 45 Gy in 25 fractions treated with two to four arcs (two arcs, eight plans; three arcs, 70 plans; four arcs, one plan). Patients were treated between 2019 and 2022 and had cancers of the cervix (27 patients), uterus (48 patients), vagina (three patients), and ovaries (one patient). The collection and use of this data were performed under protocol PA16‐1379 approved by The University of Texas MD Anderson Cancer Center Institutional Review Board. The dataset was randomly split (3:1:1) into 47 plans for training, 16 plans for validation, and 16 plans for testing. To ensure a complete contour set for each patient for model training, a validated auto‐contouring tool based on convolutional neural networks^34^ was used to generate normal tissue contours for each plan. Clinical contours were used when available with autocontours used for structures excluded from the clinical contours, most commonly for bony structures.

The data were formatted into 16 model inputs for each patient, including the computed tomography (CT) scan, target array, OARs including the body, bladder, bowel bag, rectum, left and right femoral heads, left and right kidneys, liver, and spinal cord, and bony structures including the L4 and L5 vertebral bodies, pelvic bone, and sacrum. The CT simulation scan was normalized, a common data augmentation approach for dose prediction,^35^ by cropping the voxel intensities to a range of [−1000,1000] and then rescaling to a range of [0,1]. The OARs and bony structures were input as image masks, and the target array was an image array of the planning target volume (PTV) with the values of the prescription dose. All model inputs were resampled to the dose‐grid spacing of the clinical plan (2 mm, three plans; 3 mm, 76 plans).

The model architecture was a 3D Dense Dilated U‐Net, which gave us accurate dose prediction results in our prior work on head and neck cancers.^2^, ^19^ Several custom loss functions were investigated, and model performance was compared with quantitative and qualitative evaluations of predicted dose distributions on the validation set. The top‐performing loss function was a custom mean squared error and target DVH loss function shown in Equation (1):

(1) $$\begin{array}{ccc} Loss & = & MSE\left(D_{clinical},D_{predicted}\right)+\;0.25\left[\Delta D_{1\%}\left(PTV\right)\right.\\ & & \left.+\;\Delta D_{95\%}\left(PTV\right)+\Delta D_{98\%}\left(PTV\right)\right] \end{array}$$

where $MSE(D_{clinical},D_{predicted})$ is the mean‐squared error over the entire dose distribution and $\Delta D_{1\%}\left(PTV\right),\;\Delta D_{95\%}\left(PTV\right)$, and $\Delta D_{98\%}\left(PTV\right)$ are the absolute errors in the predicted target metrics of $D_{1\%}$, $D_{95\%}$, and $D_{98\%}$, respectively, calculated using Equation (2):

(2) $$\begin{array}{c} \Delta D_{V\%}\;\left(PTV\right)=\left|D_{V\%}\left(predicted\right)-D_{V\%}\left(clinical\right)\right|\; \end{array}$$

where *V* is the volume. Models were trained using an Nvidia V100 graphical processing unit with 16GB memory. A patch‐based approach, in which patches of size (64, 64, 64) were randomly generated during training, was used. The Adam optimizer was used with an initial learning rate of 0.001. The learning rate was decreased by half after every 55 epochs of unimproved loss. Models were trained with a maximum of 1000 epochs using early stopping.

Three models were trained with the top‐performing loss function and then used to predict the test set of 16 plans. Overlapping patches were generated for each plan using a stride of 16 along each axis. All patches were predicted with each model. The plan prediction was the average of all patches across all three models. The predicted dose distributions were normalized to achieve 95% target coverage of the corresponding clinical plan using Equation (3):

(3) $$\begin{array}{c} \;D_{normalized}=D_{predicted}\;*\;\frac{D_{95\%}\left(clinical\right)}{D_{95\%}\left(predicted\right)}\; \end{array}$$

### 2.2 Comparison of predicted and clinical dose distributions

The predicted dose distributions across the test set were compared to the clinical dose distributions. To assess the equivalence of the predicted and clinical mean doses, we performed a paired two one‐sided *t*‐test under an equivalence bound of 1 Gy. Dose metrics were calculated for the PTVs and OARs. Target conformity indices, *CI*, and homogeneity indices, *HI*, were calculated for the predicted and clinical dose distributions. *CI* was defined according to Equation (4) as the ratio of the PTV covered by the reference isodose line, *TV_RI_*, and the total reference isodose volume, *V_RI_*:

(4) $$\begin{array}{c} CI\;=\;\frac{TV_{RI}}{V_{RI}} \end{array}$$

The reference isodose for the *CI* calculations was the 42.75 Gy isodose—95% of the prescription. The *HI* was defined according to Equation (5) as the ratio of $D_{5\%}\;$and $D_{95\%}\;$of the PTV:

(5) $$\begin{array}{c} HI\;=\;\frac{D_{5\%}}{D_{95\%}} \end{array}$$

The percentage dose differences in the predicted target metrics of $D_{1\%}$ and $D_{98\%}$, $\Delta D_{1\%}\left(\%\right)$ and $\Delta D_{98\%}\left(\%\right)$, were calculated using Equation (6):

(6) $$\Delta D_{V\%}\;\left(\%\right)=\frac{D_{V\%}\left(\mathrm{pred}\mathrm{icted}\right)-D_{V\%}\left(\mathrm{clin}\mathrm{ical}\right)}{D_{V\%}\left(\mathrm{clin}\mathrm{ical}\right)}\;*\;100\%$$

The dose differences in the predicted OAR mean and maximum doses, $\Delta D_{mean}$ and $\Delta D_{max}$, respectively, were calculated using Equations (7) and (8):

(7) $$\Delta \;D_{\mathrm{mean}}=D_{\mathrm{mean}}\left(\mathrm{pred}\mathrm{icted}\right)-D_{\mathrm{mean}}\left(\mathrm{clin}\mathrm{ical}\right)$$

(8) $$\Delta \;D_{\max}=D_{\max}\left(\mathrm{pred}\mathrm{icted}\right)-D_{\max}\left(\mathrm{clin}\mathrm{ical}\right)$$

### 2.3 Physician review of predicted dose distributions

The predicted dose distributions were imported into the RayStation (RaySearch Laboratories, Stockholm, Sweden) treatment planning system and reviewed by a radiation oncologist specializing in gynecologic cancers. The radiation oncologist scored the predictions using the following 5‐point scale:

5) Acceptable as‐is
4) Prefer minor edits, but I would use this plan if necessary
3) Minor edits needed
2) Major edits needed
1) Unacceptable; fails to meet clinical criteria

### 2.4 Replanning study

To determine the achievability of the predicted dose distributions and their utility in guiding plan review and optimization, a subset of five clinically treated plans from the test set was selected for a replanning study. These plans were selected because they had the largest dose differences between the predicted and clinical dose distributions with the predicted dose distributions indicating that superior sparing of normal tissues and/or OARs could have been achieved in the clinical plans. The clinical plans were manually reoptimized in RayStation by adding additional plan objectives to the original clinical plan objectives. Point objectives for max DVH and max dose were added for the OARs for which the predicted dose indicated further sparing could be achieved. DVH and dose values were selected to either match the predicted values or push slightly beyond the predicted values. To push the clinical plans toward the predicted target dose fall‐off and normal tissue sparing, normal tissue planning structures were created by subtracting the predicted isodose volumes from the body contour. Dose fall‐off and max dose plan objectives were applied to the normal tissue planning structures. The planner was allowed to perform as many optimization iterations as deemed necessary. If achieving a certain plan objective resulted in unwanted tradeoffs in target or OAR doses, the planner modified the plan objectives and weights or added additional plan objectives for the affected targets or OARs. This re‐optimization workflow was selected to mimic the clinical workflow for replanning.

## 3 RESULTS

### 3.1 Comparison of predicted and clinical dose distributions

**FIGURE 1:**
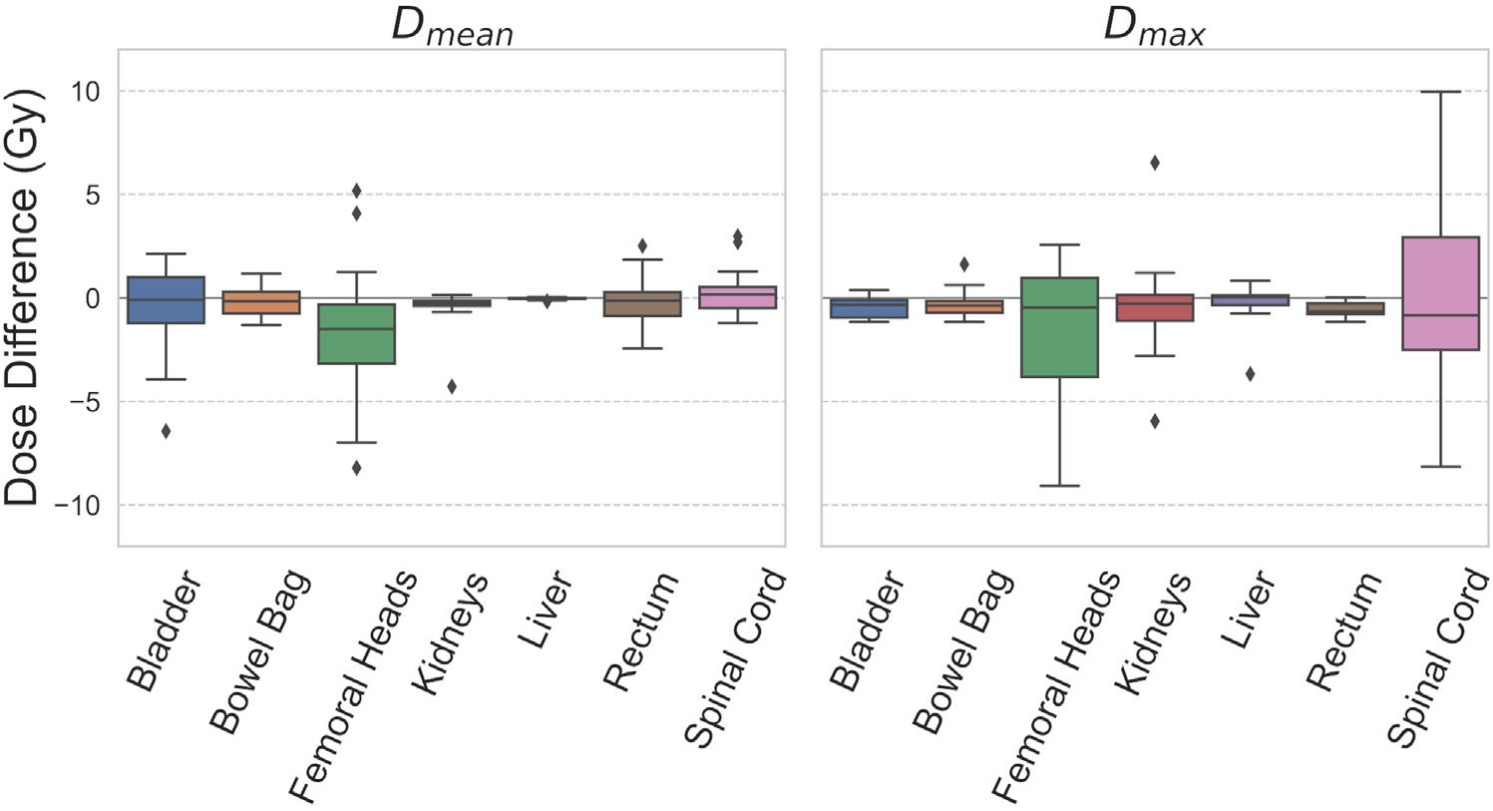
The dose differences between the predicted and clinical mean (D_mean_) and maximum (D_max_) doses for the organs at risk in the test set.

The predicted doses were compared with the clinical doses across the test set. The mean voxel‐wise dose difference was −0.14 ± 0.46 Gy. The results from our paired two one‐sided *t*‐test showed that the model‐predicted and clinical dose distributions of the test set were equivalent (*p* < 0.0001). On average, the predicted dose distributions were more conformal and homogeneous within the PTV. The mean values of *CI* for the predicted and clinical dose distributions were 0.81 ± 0.03 and 0.79 ± 0.05, respectively. The *HI* for the predicted dose distributions was 1.02 ± 0.00, and the mean value of *HI* for the clinical dose distributions was 1.03 ± 0.01. The percentage dose differences in the predicted target metrics of $D_{1\%}$ and $D_{98\%}$ were −1.05% ± 0.59% and 0.21% ± 0.28%, respectively. The dose differences in the predicted OAR mean and maximum doses were −0.30 ± 1.66 Gy and −0.42 ± 2.07 Gy, respectively. Figure 1 displays the dose differences in the predicted mean and max doses for each OAR. The largest dose differences were observed for the spinal cord max dose with dose differences ranging from −8.16 to 9.95 Gy. The two cases for which the dose difference was close to 10 Gy had clinical max doses of approximately 15 Gy and predicted max doses of approximately 25 Gy. All clinical and predicted spinal cord max doses in the test set were below 34 and 26 Gy, respectively. The large ranges in dose differences between the predicted and clinical doses for the spinal cord may be explained by the variability in treatment planning practices and the planner's persistence to push the spinal cord dose to as low as possible beyond the tolerance dose of 45 Gy.

**FIGURE 2:**
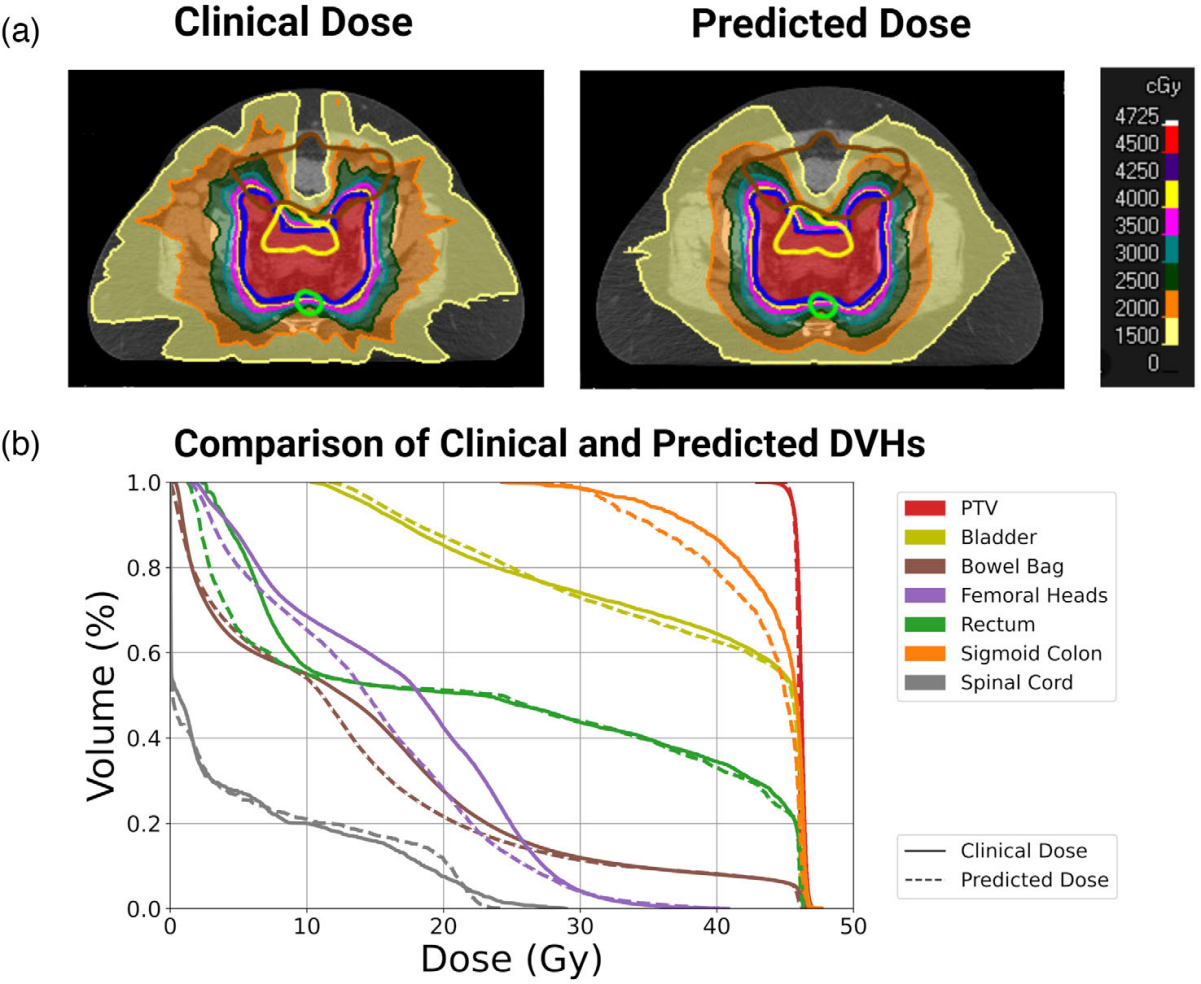
(a) Comparison of the predicted and clinical dose distributions for an example test set patient with endometrial cancer. The PTV prescribed to 45 Gy is contoured in blue. The bladder, bowel bag, and rectum are contoured in yellow, brown, and green, respectively. (b) Comparison of the predicted (dashed) and clinical (solid) dose‐volume histograms (DVHs) for the same patient.

The models accurately predicted the clinical dose distributions across the test set. Figure 2 displays an example prediction for a patient with endometrial cancer from the testing dataset. Panel a is a comparison of the predicted and clinical dose distributions on an axial slice of the CT scan. The target is contoured in blue, the bowel bag in brown, the bladder in yellow, and the rectum in green. The predicted dose distribution is conformal and homogeneous within the PTV while accurately capturing the steep dose fall‐off anteriorly and posteriorly, resulting in similar predicted doses to the bladder, bowel, and rectum as in the clinical plan. Panel b is a comparison of the DVH curves for the same patient; the clinical DVHs are shown as solid lines and the predicted DVHs are shown as dashed lines. The DVHs closely match for all structures, with the predicted dose distribution indicating that slightly better sparing of the sigmoid colon, bowel bag, and femoral heads could have been achieved in the clinical plan.

### 3.2 Physician review

All predicted dose distributions in the test set received a score of 4 (four cases) or 5 (12 cases) by the radiation oncologist. Two of the plans that were scored a 4 had poor target coverage on one slice of the CT scan, where the nodal target volumes joined. One of the plans was scored a 4 because there was a 105% hot spot in the target near the rectum. Finally, one of the plans was scored a 4 due to inferior posterior‐dose fall‐off. In this case, the skin was receiving 20 Gy, which the physician indicated might result in skin reactions.

### 3.3 Replanning study

**FIGURE 3:**
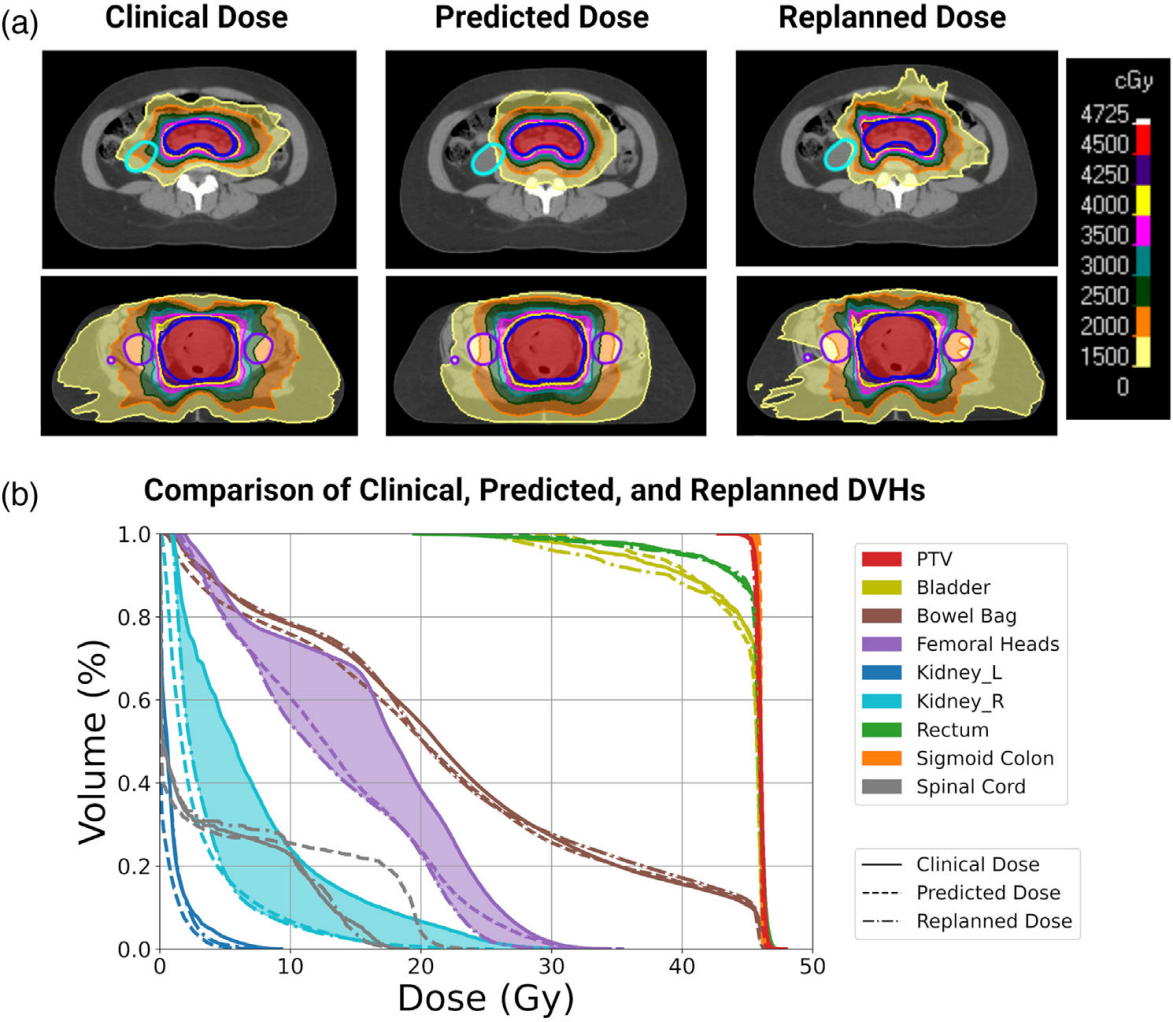
(a) Comparison of the clinical, predicted, and replanned dose distributions for an example test set patient with cervical cancer. The PTV prescribed to 45 Gy is contoured in blue. The predicted dose distribution indicated that the right kidney (contoured in aqua blue) and femoral heads (contoured in purple) could be better spared in the clinical plan. The clinical plan was re‐optimized to achieve the predicted sparing gains. (b) Comparison of the clinical (solid), predicted (dashed), and replanned (dashed‐dotted) dose‐volume histograms (DVHs) for the same patient. The improved sparing of the right kidney and the femoral heads between the clinical and replanned plans is represented by the aqua blue‐shaded and purple‐shaded DVH regions, respectively.

**FIGURE 4:**
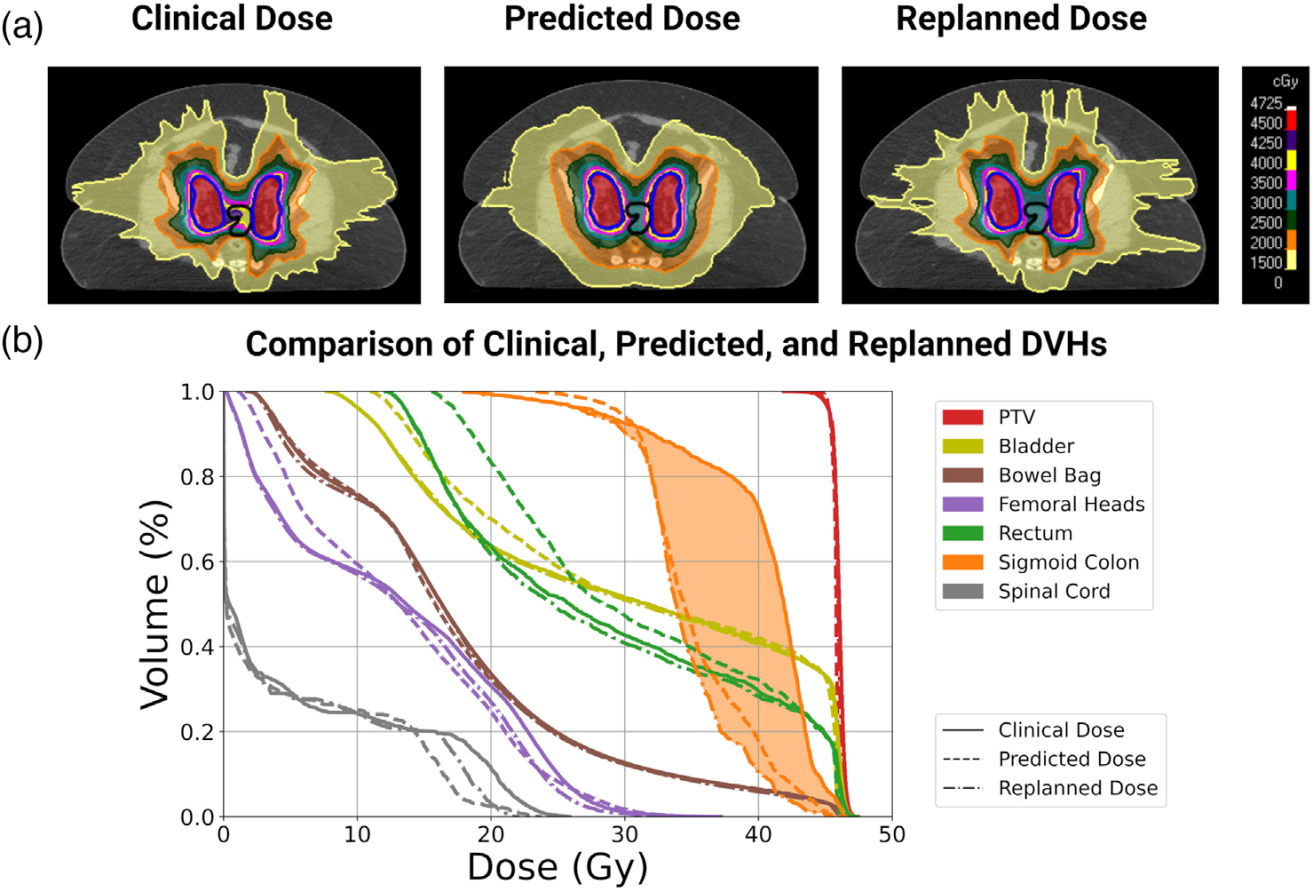
(a) Comparison of the clinical, predicted, and replanned dose distributions for an example test set patient with endometrial cancer. The PTV prescribed to 45 Gy is contoured in blue. The predicted dose distribution indicated that the sigmoid colon (contoured in black) could be better spared in the clinical plan. The clinical plan was re‐optimized to achieve the predicted sparing gains. (b) Comparison of the clinical (solid), predicted (dashed), and replanned (dashed‐dotted) dose‐volume histograms (DVHs) for the same patient. The improved sparing of the sigmoid colon between the clinical and replanned plans is represented by the orange‐shaded DVH region.

**FIGURE 5:**
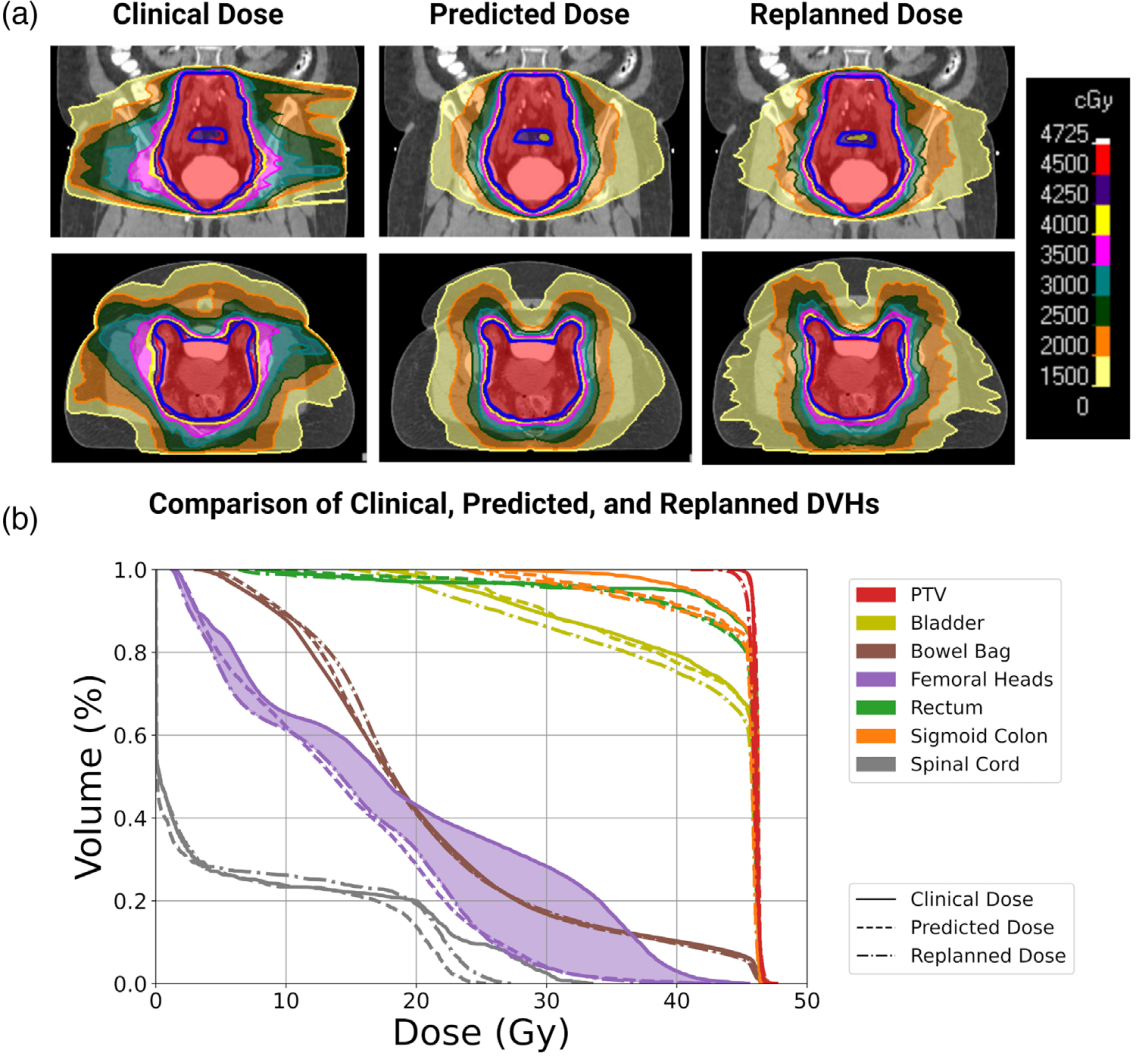
(a) Comparison of the clinical, predicted, and replanned dose distributions for an example test set patient with cervical cancer. The PTV prescribed to 45 Gy is contoured in blue. The predicted dose distribution indicated that improved sparing of the femoral heads and normal tissues could be achieved in the clinical plan. The clinical plan was re‐optimized to achieve the predicted sparing gains. (b) Comparison of the clinical (solid), predicted (dashed), and replanned (dashed‐dotted) dose‐volume histograms (DVHs) for the same patient. The improved sparing of the femoral heads between the clinical and replanned plans is represented by the purple‐shaded DVH region.

For five patients in the test set, the clinically treated plans were replanned to achieve the predicted OAR and normal tissue sparing. Dose metrics of the clinical and replans for all patients are reported in Table S1. Three of the five patients are shown in Figures 3, 4, 5. In each of these figures, Panel a is a comparison of the clinical, predicted, and replanned dose distributions and Panel b is a comparison of the clinical, predicted, and replanned DVHs represented by solid, dashed, and dashed‐dotted lines, respectively. The plan for Patient #1 with cervical cancer (Figure 3) was selected for the replanning study because the predicted dose distribution indicated that improved sparing of the right kidney (contoured in aqua blue) and femoral heads (contoured in purple) could be achieved. Replanning demonstrated that the predicted sparing of OARs was achievable while the clinical target coverage and homogeneity and sparing of the other normal tissues were maintained. The improved sparing of the right kidney and the femoral heads between the clinical and replanned plans is represented by the aqua blue‐shaded and purple‐shaded DVH regions, respectively.

The plan for Patient #2 with endometrial cancer (Figure 4) was selected for the replanning study because the predicted dose indicated that further sparing of the sigmoid colon could be achieved in the clinical plan. As shown in Panel a, the sigmoid colon (contoured in black) is between the target volumes (contoured in blue). Our model predicted that superior dose fall‐off between the targets and the sigmoid colon could have been achieved in the clinical plan. Replanning demonstrated that the predicted sparing benefits were achievable. Replanning reduced the sigmoid *V_40 Gy_* from 72% to 8%. The improved sigmoid colon dose sparing is represented by the orange‐shaded DVH region in Panel b.

Patient #3 in the replanning study was selected because the predicted dose distribution indicated that better dose fall‐off from the target and reduced normal tissue dose could have been achieved in the clinical plan. The clinical plan for this patient was observed to be an outlier and unrepresentative of typical plans in our clinic which have more conformal dose distributions. The clinical, predicted, and replanned dose distributions for this patient, who had cervical cancer, are displayed in Figure 5. The replanned dose distribution has improved target conformity; the *CI* increased from 0.78 to 0.87 with re‐optimization of the clinical plan. Moreover, the replanned dose distribution has a much sharper dose gradient from 40 to 20 Gy than the clinical plan. Aside from the predicted sparing gains for the femoral heads, the predicted OAR sparing for this plan was similar to the clinical plan, highlighting the importance of 3D dose predictions in capturing further plan improvements for normal tissues that might not be caught in a DVH prediction. For example, the dose to the abdominal pannus is significantly lower in the predicted and replanned doses than the clinical dose. Had the predicted dose distribution been used for prospective plan quality assessment, the clinical dose distribution would have been flagged for risk of patient skin reactions.

## 4 DISCUSSION

In this study, we developed a dose‐prediction model that predicted high‐quality 3D dose distributions for VMAT plans for the female pelvis, all of which were deemed to be clinically acceptable by a radiation oncologist specializing in gynecologic cancers. Through a retrospective planning study, we showed that the predicted dose distributions can be used to improve clinical plans. To our knowledge, this is the first study to include a physician review of 3D dose distributions generated by a deep‐learning algorithm and to demonstrate the potential clinical gains of using 3D dose prediction to guide plan review and optimization.

Although the predicted 3D dose distributions from our model are not deliverable plans because they do not include any planning parameters, they were trained using real treatment plans and give distributions that are in good agreement with the real plans. Additionally, the results of the replanning study suggest that our model's dose predictions can be physically achieved. In this work, the predictions were used to guide manual replanning. However, the predictions could be used in a more systematic and automatic way. In our previous work, we showed the ability of dose prediction to automatically flag suboptimal plans.^2^ Once suboptimal plans are identified, replanning could be done automatically through the auto‐creation of planning objectives from predicted DVH points—similar to other knowledge‐based planning approaches—as well as predicted isodose volumes. In our manual replanning study, we found that creating planning structures from the predicted isodose volumes allowed us to take advantage of the 3D information and to achieve the predicted dose fall‐off, especially for normal tissues not classified as OARs.

During the physician review of the predicted dose distributions, the smooth nature of the predicted isodose lines was noted as being uncharacteristic of clinical dose distributions. The largest differences between the clinical and predicted dose maps were in the low‐dose regions. The 15 Gy isodoses varied greatly across the clinical plans in the training and validation sets. This variation may be the result of the low doses being of less focus during planning as there are no clear planning goals for mid‐low dose falloff. Because of the large variability in the clinical isodoses, our deep learning model was unable to identify patterns related to the planning inputs and the 15 Gy isodoses, and it likely averaged the isodoses over the training set.

In the replanning study, we showed that our patient‐specific estimates of optimal 3D dose distributions could have resulted in improved clinical plans. The plans used in this study were retrospective clinical plans, meaning that they had passed physician review. Our findings demonstrate that suboptimal plans do unfortunately make it through peer review undetected and reach patients and highlight the potential benefits of incorporating data‐driven approaches to bring plans from high quality to best possible for a given patient. This tool could be used as part of the treatment planning process to improve plans before physician review, thereby reducing the number of handoffs between the planner and physician as well as reducing inter‐planner variability. A physics reviewer or physician peer reviewer could also use it as a plan review tool. Furthermore, it has the potential to be used in an educational environment to train less‐experienced members of a radiation oncology team to achieve high‐quality plans.

## 5 CONCLUSIONS

In summary, we have demonstrated that deep learning can predict high‐quality dose distributions for VMAT plans for patients with gynecologic cancers, and that these dose distributions can be used to demonstrate achievable improvements in clinical plans.

## CONFLICT OF INTEREST STATEMENT

The Radiation Planning Assistant Project Team receives funding from the NCI, Wellcome Trust, CPRIT, Varian Medical Systems, and MD Anderson Cancer Center.

## Supporting information

Supplementary Information
