# Supplementary material for: Deep learning–based dose prediction to improve the plan quality of volumetric modulated arc therapy for gynecologic cancers
Source: Med Phys. 2023 Sep 14;50(11):6639–48. doi: 10.1002/mp.16735 (PMC10947338; doi:10.1002/mp.16735)
Supplement: Supplementary file 1 — Supplementary Information [file MP-50-6639-s001.docx]

**Table S-1.** Comparison of dose metrics for the clinically treated plans and replans for the five patients in the replanning study. The clinically treated plans were replanned to achieve predicted OAR and normal tissue sparing.

| **Structure** | **Metric** | **Patient #1 Clinical Plan** | **Patient #1 Replan** | **Patient #2 Clinical Plan** | **Patient #2 Replan** | **Patient #3 Clinical Plan** | **Patient #3 Replan** | **Patient #4 Clinical Plan** | **Patient #4 Replan** | **Patient #5 Clinical Plan** | **Patient #5 Replan** |
| --- | --- | --- | --- | --- | --- | --- | --- | --- | --- | --- | --- |
| **PTV** | *D_max_/D_Rx_* (%) | 105.49% | 106.84% | 105.58% | 106.20% | 105.93% | 105.84% | 105.16% | 105.49% | 107.51% | 106.16% |
|  | *V_95%_* (%) | 99.99% | 100.00% | 99.94% | 99.88% | 100.00% | 99.90% | 99.99% | 99.89% | 99.97% | 99.98% |
|  | *CI* | 0.83 | 0.83 | 0.82 | 0.85 | 0.78 | 0.87 | 0.77 | 0.82 | 0.80 | 0.81 |
|  | *HI* | 1.02 | 1.02 | 1.03 | 1.03 | 1.02 | 1.03 | 1.02 | 1.03 | 1.03 | 1.02 |
| **Bladder** | *D_max_/D_Rx_* (%) | 104.38% | 104.22% | 105.51% | 105.53% | 104.51% | 104.89% | 104.24% | 104.56% | 106.27% | 104.64% |
|  | *V_45Gy_* (%) | 76.56% | 76.04% | 32.97% | 33.05% | 68.33% | 64.72% | 62.20% | 54.11% | 45.79% | 44.99% |
|  | *V_40Gy_* (%) | 89.74% | 87.53% | 40.88% | 40.40% | 79.26% | 75.13% | 77.59% | 67.48% | 54.10% | 52.98% |
|  | *V_30Gy_* (%) | 97.98% | 95.95% | 51.40% | 50.89% | 88.85% | 86.05% | 93.60% | 80.94% | 64.78% | 63.35% |
| **Bowel Bag** | *D_max_/D_Rx_* (%) | 105.00% | 105.31% | 105.58% | 105.53% | 105.22% | 105.29% | 104.93% | 105.09% | 106.22% | 105.18% |
|  | *V_40Gy_* (%) | 15.69% | 17.23% | 6.54% | 6.03% | 10.51% | 9.83% | 2.59% | 2.26% | 7.95% | 7.77% |
|  | *V_40Gy_* (cc) | 325.24cc | 357.19cc | 206.18cc | 190.09cc | 222.31cc | 208.06cc | 28.56cc | 24.83cc | 155.66cc | 152.05cc |
|  | *V_30Gy_* (cc) | 565.62cc | 576.10cc | 407.37cc | 394.10cc | 361.75cc | 374.69cc | 58.87cc | 51.87cc | 272.99cc | 271.82cc |
| **Left Femoral Head** | *D_max_* (Gy) | 35.81Gy | 34.91Gy | 40.66Gy | 40.00Gy | 45.09Gy | 42.54Gy | 41.33Gy | 38.89Gy | 43.58Gy | 44.05Gy |
|  | *V_45Gy_* (%) | 0.00% | 0.00% | 0.00% | 0.00% | 0.00% | 0.00% | 0.00% | 0.00% | 0.00% | 0.00% |
|  | *V_40Gy_* (%) | 0.00% | 0.00% | 0.00% | 0.00% | 0.89% | 0.02% | 0.03% | 0.00% | 0.10% | 0.10% |
| **Right Femoral Head** | *D_max_* (Gy) | 38.44Gy | 33.90Gy | 38.00Gy | 37.22Gy | 46.05Gy | 44.39Gy | 41.22Gy | 37.38Gy | 38.80Gy | 37.60Gy |
|  | *V_45Gy_* (%) | 0.00% | 0.00% | 0.00% | 0.00% | 0.27% | 0.00% | 0.00% | 0.00% | 0.00% | 0.00% |
|  | *V_40Gy_* (%) | 0.00% | 0.00% | 0.00% | 0.00% | 4.97% | 0.36% | 0.00% | 0.00% | 0.00% | 0.00% |
| **Left Kidney** | *D_mean_* (Gy) | 0.92Gy | 0.84Gy | 0.40Gy | 0.33Gy | 0.75Gy | 0.72Gy | 0.06Gy | 0.05Gy | 0.02Gy | 0.02Gy |
|  | *V_20Gy_* (%) | 0.00% | 0.00% | 0.00% | 0.00% | 0.00% | 0.00% | 0.00% | 0.00% | 0.00% | 0.00% |
|  | *V_15Gy_* (%) | 0.00% | 0.00% | 0.00% | 0.00% | 0.00% | 0.00% | 0.00% | 0.00% | 0.00% | 0.00% |
| **Right Kidney** | *D_mean_* (Gy) | 7.36Gy | 3.52Gy | 0.30Gy | 0.24Gy | 1.69Gy | 1.56Gy | 0.13Gy | 0.10Gy | 0.22Gy | 0.22Gy |
|  | *V_20Gy_* (%) | 6.70% | 0.01% | 0.00% | 0.00% | 0.00% | 0.00% | 0.00% | 0.00% | 0.00% | 0.00% |
|  | *V_15Gy_* (%) | 12.74% | 2.16% | 0.00% | 0.00% | 0.03% | 0.03% | 0.00% | 0.00% | 0.00% | 0.00% |
| **Rectum** | *D_max_/D_Rx_* (%) | 104.98% | 104.93% | 105.24% | 104.24% | 104.29% | 104.80% | 103.51% | 103.89% | 106.49% | 105.13% |
|  | *V_45Gy_* (%) | 86.86% | 87.88% | 18.88% | 19.08% | 86.98% | 81.63% | 58.51% | 54.77% | 40.79% | 40.41% |
|  | *V_40Gy_* (%) | 95.27% | 94.84% | 29.00% | 27.80% | 94.69% | 90.97% | 79.33% | 74.20% | 45.95% | 45.73% |
|  | *V_30Gy_* (%) | 98.86% | 98.61% | 42.04% | 40.21% | 95.93% | 95.38% | 87.51% | 85.27% | 54.80% | 55.12% |
| **Sigmoid Colon** | *D_max_/D_Rx_* (%) | 103.78% | 104.18% | 103.71% | 101.71% | 104.02% | 103.56% | 104.40% | 103.91% | 105.71% | 104.24% |
| **Spinal Cord** | *D_max_* (Gy) | 18.97Gy | 19.15Gy | 25.88Gy | 23.96Gy | 35.37Gy | 28.59Gy | 22.36Gy | 21.16Gy | 27.31Gy | 24.19Gy |
